# Machine learning-based screening of heart failure using the integrated features of electrocardiogram and phonocardiogram: a multicenter study in China

**DOI:** 10.3389/fcvm.2025.1613577

**Published:** 2025-11-21

**Authors:** Junjie Bian, Kok-Han Chee, Chengyu Liu, Hongwei Sun, Shixi Zhang, Peili Chen, Hua-Nong Ting

**Affiliations:** 1 Institute of Tibetan Medicine, University of Tibetan Medicine, Lhasa, China; 2 Department of Biomedical Engineering, Faculty of Engineering, Universiti Malaya, Kuala Lumpur, Malaysia; 3 Department of Medicine, Faculty of Medicine, Universiti Malaya, Kuala Lumpur, Malaysia; 4 School of Instrument Science and Engineering, Southeast University, Nanjing, Jiangsu, China; 5 Department of Cardiology, Hefei BOE Hospital, Hefei, Anhui, China; 6 Department of Infectious Disease, Shangqiu Municipal Hospital, Shangqiu, Henan, China; 7 Intensive Care Unit, The First People’s Hospital of Shangqiu City, Shangqiu, Henan, China; 8 Faculty of Medical Engineering, Jining Medical University, Jining, Shandong, China

**Keywords:** heart failure, phonocardiogram, electrocardiogram, machine learning, prediction model

## Abstract

**Backgrounds:**

Heart failure (HF) is a major health concern associated with poor prognosis, and there is an urgent clinical need for an easy and accurate method for screening HF. This multicenter study aims to validate a novel AI-based phono-electrocardiogram algorithm (AI-PECG) in early HF detection.

**Methods:**

A total of 1,017 individuals were grouped into a training cohort and an external validating cohort, with a ratio of 8:2. In the training cohort, data of patients were further split into training set and test set randomly with the 8:2 ratio. The least absolute shrinkage and selection operator with five-fold cross-validation was utilized for dimensionality reduction and selection of features for model construction from clinical variables, phonocardiogram (PCG) parameters and electrocardiogram (ECG) parameters. Five machine learning (ML) algorithms were then carried out to choose a classifier model with the optimal recognition of HF, including logistic regression, random forest, eXtreme Gradient Boosting, Category Boosting (CatBoost), and Naive Bayes. The importance of ranking predicted factors was calculated in the final screening model using the SHapley Additive exPlanations analysis.

**Results:**

Among eligible participants, 302 reported HF. Totally 17 variables were selected to conduct the screening models. In the training set, the area under the curve (AUC) of the CatBoost model was 0.998 [95% confidence interval (CI): 0.996–1.000], which was higher compared to that of other ML models. The sensitivity and specificity of CatBoost model was 0.989 (95% CI: 0.978–0.996) and 0.989 (95% CI: 0.979–0.999). In the screening model, top 5 factors in terms of importance were EMAT, lymphocyte, LVST, CRP, and platelet.

**Conclusion:**

The ML model incorporating general data alongside ECG and PCG features carried out good detection performance for HF. This had the potential to be an available tool for clinicians to screen HF patients as early as possible for further clinical interventions.

## Introduction

Heart failure (HF), compounded by late diagnosis, remains a major contributor to high morbidity and mortality (1, 2). In China, nearly 12.1 million people are affected by HF, with approximately 3 million new cases each year (3). Despite new medical therapy improved clinical outcomes for patients with HF, the 5-year mortality rate is still nearly 50% (4). Early detection of HF can delay the progression and improve long-term prognosis (5). The 12-lead electrocardiogram (ECG) and phonocardiogram (PCG) are common initial screening tools for cardiac disease in clinical practice due to their rapid, simple, and non-invasive nature, providing important insights into heart structure and hemodynamic parameters (6, 7). However, the early-stage symptoms and signs of HF often show insufficient sensitivity and specificity when screened only using ECG or PCG (8, 9), leading to a limited accuracy (10). Therefore, integrating ECG and PCG signals may offer clinical value in detecting complex cardiac diseases (11), which may have potential clinical value in the detection of HF in untested populations.

Advancements in artificial intelligence (AI) technology are being utilized to detect cardiovascular diseases through biomedical signal analysis (12–14). A prospective, observational, multicenter study in the UK indicated that AI-ECG have the potential to be inexpensive, noninvasive, and workflow-adapted for earlier HF detection (14). This inspired a novel approach that combines AI algorithm with the integrated features of heart sounds and cardiac electrical activity, which enables interpreting any adequate quality ECG and PCG signals and produces a prediction model for HF diagnosis. AI-based phono-electrocardiogram algorithm (AI-PECG) is a new technique that utilizes AI algorithms to collect and analyze the signals of cardiac electrical activity and heart sounds simultaneously during routine auscultation. It can create a graph of cardiac electrical activity and heart sound murmurs by using a miniature sensor during a heart cycle, offering an earlier detection reference for complex heart diseases (15). The emergence of AI-PECG presents an opportunity to leverage the combined features of ECGs and PCGs for simultaneous initial screening of HF, while also facilitating the development of a screening tool based on machine learning (ML) models by using the combined features. Tools based on ML models for screening cardiac diseases have been developed (16, 17), but those specifically designed for HF remain limited.

Therefore, we developed ML-based HF detection models by using ECG and PCG parameters as well as conventional HF risk factors. The study aims to arrive at a final model that outperforms the existing HF screening model through different ML-based models trained and tested on cohorts and to validate the potential of AI-PECG for HF early detection.

## Method

### Study design and population

This is a multicenter, retrospective cohort study designed to construct and evaluate an HF detection model reliant on electronic health records and PECG data. A total of 1,017 patients received PECG examination in three hospitals in two provinces of China between January 2023 and December 2023 were recruited. Exclusion criteria based on age or diagnosed disorder were applied, meaning that patients aged ≥18 years, without HF history and other severe heart disease history that will influence the interpretation of ECG or PCG were included. Also, patients with incomplete AI-PECG features and missing important health record data were excluded because their records were not suitable for the training and test model. HF was diagnosed based on the American College of Cardiology (ACC) and American Heart Association (AHA) guidelines for the management of HF (18).

The independent reviewer extracted patients’ demographic information, medical contact details, and final diagnoses from electronic health records, and the features of both ECG and PCG were identified from the AI-PECG system. The AI-PECG features as well as health records data were merged by the unique ID of patients. Adjudications were made by independent reviewers at each local site after reviewing all available medical records, and the reviewers were blinded from all feature analyses and models’ predictions.

### ECG and PCG parameters management

The parameters of ECG and PCG were acquired from patients upon their initial contact with the hospital using AI-PECG devices. Patients assumed a quiet supine position for approximately 5–10 min, maintaining stable respiration throughout. The AI-PECG devices were connected to the chest and limb leads following the conventional 12-lead ECG method, with V3 and V4 leads positioned with dual receptors for both ECG and PCG, allowing synchronous recording of signals for a duration of 2 min. Each patient had at least three consecutive records obtained. Digital PECG files were exported in.xml format and stored on a secondary server at each local site. AI-PECG images were de-identified and manually annotated by independent reviewers or research specialists. AI-PECG recordings with poor quality or missing leads were excluded. Subsequently, digital (with.xml format) files were analyzed offline.

### Features selection

Participants were randomly divided into a training cohort and an external validating cohort first, with an 8:2 ratio. Then, data of patients in the training cohort were further split into training sets and test sets randomly also with the 8:2 ratio. All feature selection processes were conducted within the training set. To mitigate omitted feature bias, we adopted a data-driven approach [5-fold least absolute shrinkage and selection operator (LASSO)] for feature selection. Initially, features of conventional risk factors [including sex, age, hypertension, hypotension, coronary artery disease (CAD), heart rate (HR), hemoglobin (HB), lymphocyte, platelet, total cholesterol (TC), triglyceride (TG), C-reactive protein (CRP)], PCG parameters [electro mechanical activation time (EMAT)/left ventricular systolic time (LVST), EMAT (%, the EMAT to RR ratio), LVST, first heart sound (S1), second heart sound (S2), third heart sound (S3), and fourth heart sound (S4)], and ECG parameters [including Axes, *P* wave duration (PD), PR interval duration (PRD), QRS complex duration (QRSD), QT interval duration (QTD), V5 lead R-wave amplitude plus V1 lead S-wave amplitude (RV5_SV1)] were all included. S1 is the first sound in the heart sound cycle, indicating the beginning of ventricular contraction and is produced by the closure of the mitral and tricuspid valves due to the pressure difference between the atria and ventricles. S2 indicates the beginning of the ventricular diastole and is mainly generated by the vibrations when the aortic valve and pulmonary valve close. The EMAT is the time between the Q peak and the beginning of the S1 signal. The LVST indicates the time duration between the peak of the S1 sound and that of S2 sound. Subsequently, the LASSO with five-fold cross-validation was employed for dimensionality reduction and selection of these features. The final variables used for model construction were selected based on the smallest mean square error (MSE) for each penalty coefficient λ.

### Prediction modeling and evaluation

**Figure 1:**
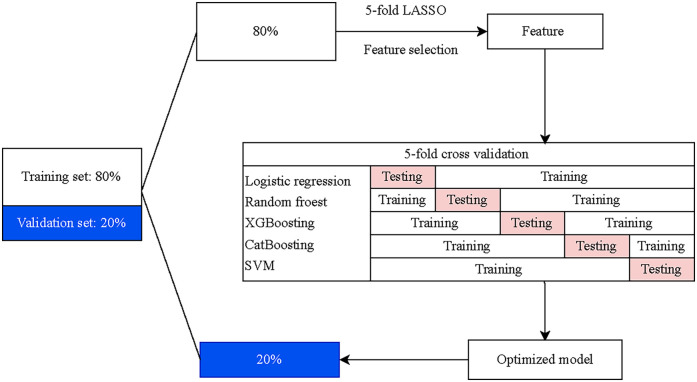
The flow chat of model development and validation.

Five ML algorithms were carried out to choose a classifier model with the optimal recognition of HF, including logistic regression (LR), random forest (RF), eXtreme Gradient Boosting (XGBoost), Category Boosting (CatBoost), and Naive Bayes (NB). Features for model construction were selected utilizing the five ML algorithms with the five-fold cross-validation. In the training set, data were divided into five subsets, four of which were served as the training set and the other as the validation set. Five iterations were then performed, and the means of the cross-validations and the best performance fold were taken as the final classification results to screen the optimal screening model. The screening ability of the final model was further validated by the test set. The flow chart of model development and validation is presented in Figure 1.

### Model interpretation

To evaluate the prediction value and accuracy of various ML models, we calculated and compared areas under the curve (AUC) of the receiver operating characteristic curve (ROC), sensitivity, and specificity. The SHapley Additive exPlanation (SHAP) values were used to provide consistent and locally accurate attribution values for each feature within each prediction model, which is a unified approach for explaining the outcome of any ML model. All SHAP values were computed using the training set.

### Statistical analysis

All statistical analyses were performed using R software (version 4.3.3, R Foundation for Statistical Computing, Vienna, Austria). Continuous data were presented as mean ± standard deviation (SD) and categorical data were presented as numbers with percentages [n (%)]. Differences in continuous data were compared using the t-test or Wilcoxon rank-sum test, and differences in categorical data were compared using the *χ*^2^ test or Fisher's exact test. The fitting of the final model was evaluated by plotting ROC curves, calibration curve, and decision curve analysis (DCA) curves. The importance of ranking predicted factors was calculated in the final screening model using the SHAP analysis (shapviz package available on CRAN). The correlations of the detection factors with HF were further assessed. *P*-value < 0.05 was considered as the statistical significance.

## Results

### Characteristics of participants

Among 1,017 eligible patients, 302 patients reported HF (Table 1). Of the overall samples, the mean age was 68.16 (10.44) years and 523 (51.43%) were female; 326 (32.06%) had hypertension and 522 (51.33%) had CAD; 432 (42.48%) reported S3 heart sound and 88 (8.65%) reported heart sound S4. Compared to non-HF group, the HF group had higher levels of HR (74.44 vs. 71.02, *P* < 0.001), CRP (9.88 Mg/L vs. 7.93 Mg/L, *P* < 0.001), EMAT/LVST (0.40 vs. 0.30, *P* < 0.001), EMAT (13.97 ms vs. 10.84 ms, *P* < 0.001), S2 (48.36 ms vs. 41.31 ms, *P* < 0.001), PRD (142.51 vs. 131.57, *P* = 0.021), QRSD (82.87 vs. 77.45, *P* = 0.024) and RV5_SV1 (7.34 vs. 1.68, *P* < 0.001). No significant difference has been observed between these two groups in other characteristics (all *P* > 0.05).

**Table 1:** Patients’ characteristics included in the HF screening model.

Variables	All (*n* = 1,017)	non-HF (*n* = 715)	HF (*n* = 302)	*P*-value
Sex, *n* (%)				0.565
Male	494 (48.57)	352 (49.23)	142 (47.02)	
Female	523 (51.43)	363 (50.77)	160 (52.98)	
Age, Mean (SD)	68.16 (10.44)	68.60 (10.37)	67.11 (10.55)	0.115
Hypertension, *n* (%)	326 (32.06)	226 (31.61)	100 (33.11)	0.692
Hypotension, *n* (%)	147 (14.45)	101 (14.13)	46 (15.23)	0.718
CAD, *n* (%)	522 (51.33)	375 (52.45)	147 (48.68)	0.303
HR, mean (SD)	72.03 (13.13)	71.02 (12.64)	74.44 (13.97)	<0.001
Biochemical indicators, mean (SD)				
HB, g/L	124.12 (21.35)	124.21 (20.84)	123.89 (22.54)	0.977
Lymphocyte, 10 /L	1.59 (0.66)	1.59 (0.75)	1.60 (0.37)	0.976
Platelet, 10 /L	169.68 (21.98)	170.00 (23.32)	168.94 (18.43)	0.782
TC, mmol/L	3.56 (1.49)	3.57 (1.41)	3.54 (1.68)	0.954
TG, mmol/L	1.09 (0.34)	1.09 (0.33)	1.09 (0.35)	0.984
CRP, Mg/L	8.51 (6.55)	7.93 (6.23)	9.88 (7.06)	<0.001
PCG, mean (SD)				
EMAT/LVST	0.33 (0.07)	0.30 (0.05)	0.40 (0.08)	<0.001
EMAT, ms, mean (SD)	11.77 (3.10)	10.84 (2.32)	13.97 (3.57)	<0.001
LVST, ms, mean (SD)	0.33 (0.07)	0.30 (0.05)	0.40 (0.08)	0.157
S1, ms	50.91 (23.61)	51.08 (22.01)	50.50 (27.05)	0.938
S2, ms	43.41 (26.03)	41.31 (23.58)	48.36 (30.56)	<0.001
Visibility of S3, *n* (%)	432 (42.48)	254 (35.52)	178 (58.94)	<0.001
Visibility of S4, *n* (%)	140 (13.77)	100 (13.99)	40 (13.25)	0.831
Visibility of S3 and S4, *n* (%)	88 (8.65)	57 (7.97)	31 (10.26)	0.286
ECG, mean (SD)				
Axes	32.90 (52.14)	34.39 (49.49)	29.36 (57.87)	0.372
PD	88.11 (35.23)	87.21 (37.03)	90.24 (30.47)	0.455
PRD	134.8 (57.58)	131.57 (60.01)	142.51 (50.64)	0.021
QRSD	79.06 (29.03)	77.45 (29.35)	82.87 (27.93)	0.024
QTD	407.22 (43.61)	406.42 (39.69)	409.11 (51.73)	0.668
RV5_SV1	3.36 (19.98)	1.68 (0.72)	7.34 (36.37)	<0.001

HF, heart failure; SD, standard deviation; HR, heart rate; Hb, hemoglobin; TC, total cholesterol; TG, triglyceride; CRP, C-reactive protein; CAD, coronary artery disease; EMAT, electro mechanical activation time; LVST, left ventricular systolic time; S1, first heart sound; S2, second heart sound; S3, third heart sound; S4, fourth heart sound; PD, P wave duration; PRD, PR interval duration; QRSD, QRS complex duration; QTD, QT interval duration; RV5 SV1, V5 lead R-wave amplitude plus V1 lead S-wave amplitude.

### Model construction with different machine learning methods

**Figure 2:**
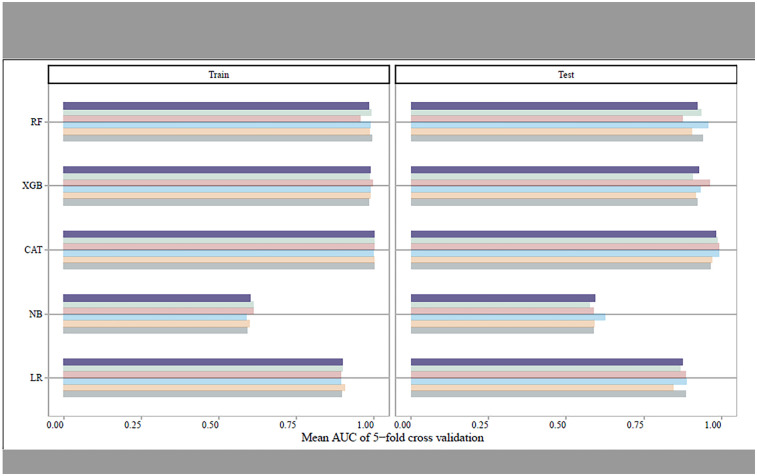
The mean AUCs of the five-fold cross-validation for different machine learning models in training set and test set, respectively.

Utilizing the Five-fold cross-validation approach, we identified 17 predictors comprising four conventional risk factors (age, CRP, HR, HB), seven PCG features (EMAT, LVST, S1 heart sound, S2 heart sound, S3 heart sound, S4 heart sound, S3 and S4 heart sound), and six ECG features (QTD, PRD, PD, RV5_SV1, QRSD, Axes) for construction of the screening models (Table 1). Figure 2 showed the performance of different ML classifiers in detection HF within the training and test datasets, respectively. According to the mean values of AUC of 5-fold cross validation, the CAT classifier exhibited the best performance, demonstrating robust generalizability to both the training set and the test set.

### Model validating and explainability

**Figure 3:**
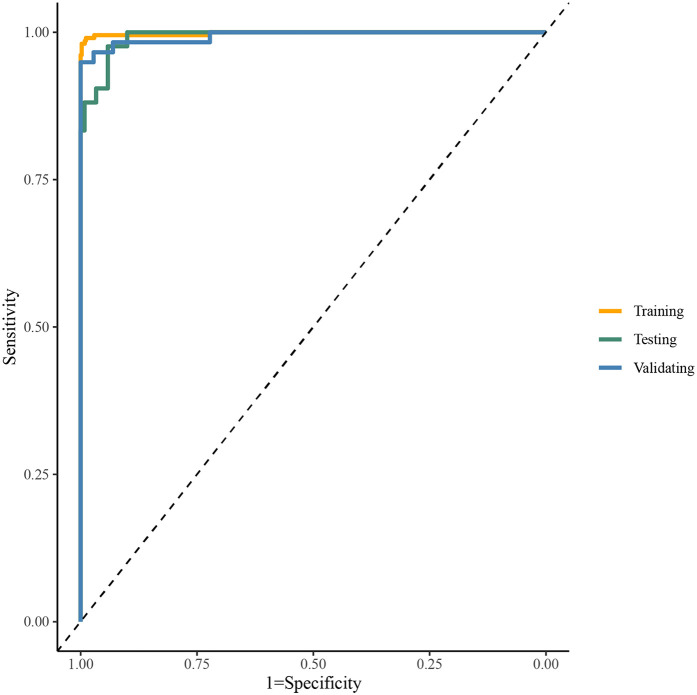
The ROC curve of the catBoost model.

Table 2 presented the performance of the CatBoost model in screening HF across the training, test, and validating datasets. The AUCs of the CatBoost model in the training and test sets were 0.998 (95%CI: 0.996–1.000) and 0.992 (95%CI: 0.984–1.000), respectively. In the validating dataset, the AUC of the CatBoost model was 0.994 (95%CI: 0.984–1.000). The sensitivity of the model in the training set, test set and validating set was 0.989 (95%CI: 0.979–0.999), 0.958 (95%CI: 0.923–0.994) and 0.972 (95%CI: 0.945–0.999) respectively (Figure 3). The specificity of the model in the training set, test set and validating set was 0.990 (95%CI: 0.977–1.000), 0.905 (95%CI: 0.816–0.994), and 0.966 (95%CI: 0.920–1.000), respectively. The fitting of the final model was illustrated by calibration curves (Supplementary Figure S1) and DCA curves (Supplementary Figure S2). Moreover, comparation on mean AUCs between the final model and model that was constructed by ECG and PCG features showed a similar performance on HF detection (Supplementary Figure S3).

**Table 2:** The detection performance of the catBoots model.

Sets	AUC	Accuracy	Sensitivity	Specificity	PPV	NPV
Training set	0.998 (0.996–1.000)	0.989 (0.978–0.996)	0.989 (0.979–0.999)	0.990 (0.977–1.000)	0.995 (0.989–1.000)	0.976 (0.956–0.997)
Test set	0.992 (0.984–1.000)	0.944 (0.897–0.974)	0.958 (0.923–0.994)	0.905 (0.816–0.994)	0.966 (0.934–0.999)	0.884 (0.788–0.980)
Validation set	0.994 (0.984–1.000)	0.970 (0.937–0.989)	0.972 (0.945–0.999)	0.966 (0.920–1.000)	0.986 (0.967–1.000)	0.934 (0.872–0.997)

AUC, the area under the curve; PPV, positive prediction value; NPV, negative prediction value.

**Figure 4:**
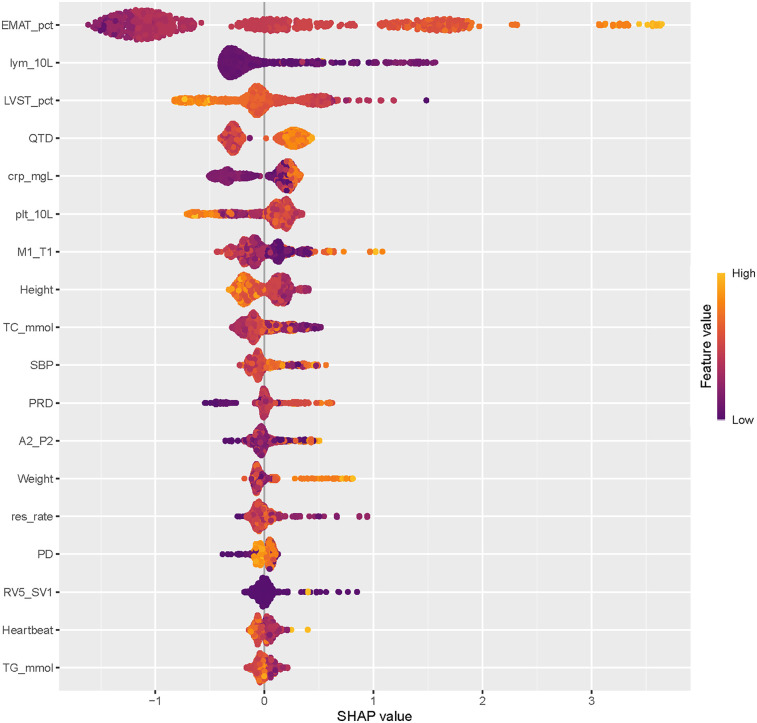
The rank of the importance of features in the catBoost model for HF screening.

**Figure 5:**
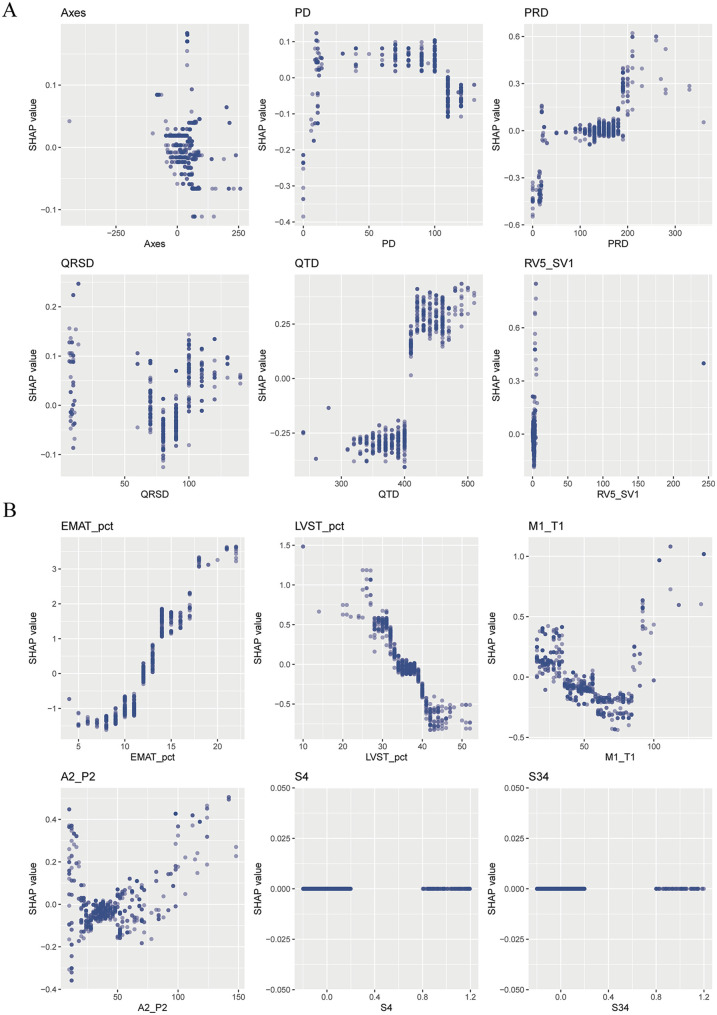
The SHAP dependence plot of the catBoost model. **(A)** ECG parameters; **(B)** PCG parameters.

The SHAP summary plot of CatBoost showed the most influential features in the final screening model, revealing that the top 5 important features were EMAT, lymphocyte, LVST, CRP, and platelet (Figure 4). This plot illustrated the relationship between feature values and SHAP values in the training dataset, with higher SHAP values indicating a greater likelihood of HF. Additionally, the SHAP dependence plot offered insight into how individual ECG features (Figure 5A) and PCG features (Figure 5B) impact the CatBoost model's output. This visualization demonstrated how the attributed importance of a feature changes as its value fluctuates.

## Discussion

To our knowledge, this was the first clinical study that validated and tested the performance of ML-based models to detect HF using the simultaneous features of PCG and ECG collected from AI-PECG. The ML-based HF detection model was trained and validated on 1,017 participants from three hospitals demonstrated a strong identification performance, with an AUC of 0.998, a sensitivity of 0.989, and a specificity of 0.990. These findings indicated that this model combined features of PCG and ECG with conventional risk factors have the potential to apply in early screening of HF in clinical. Moreover, this model was dominated by relatively few predictors, making it possible to predict with very high and fast detection based on only a few predictors.

According to the study results, using a clinical detection support tool based on the simultaneous features of PCG and ECG, when combined with the conventional risk factors, could be imperative for improving the accuracy of detecting HF. This estimate was quite similar to the current literature that used the joint data of PCGs, ECGs, and conventional risk factors to predict cardiac diseases (19–22). However, the development of such models in HF is still limited due to the absence of relevant datasets for training and validation. In the existing algorithms, most of them have only used ECGs or electronic health records, and few studies have applied PCGs. The accuracy range of the existing algorithm by only using ECGs for predicting HF was about 80.0%–98.9% (23–25), while the models using electronic health record with a sensitivity of 83%–95.3% (26, 27). For the models using PCGs, the accuracy was about 82.6%–88.2% (28). Although some of these models performed well with high AUC and sensitivity, the size and nature of these databases limited their application to clinical practice. The major challenge in the clinical application of ECG or PCG to HF detection may be that the abnormal symptoms of patients are inconspicuous or even absent in some cases. As the first study to use a ML approach for HF detection, our findings indicated that joint analysis of ECG and PCG could be a good solution to the above issue since ECG and PCG signals can reflect the electrical and mechanical activities of the heart respectively, which provides more reliable and complete evidence for early detection.

Furthermore, SHAP values were used to uncover the black box of ML and to facilitate the model interpretation. In the present study, the top 5 most influential features contributing to this model were EMAT, lymphocyte, LVST, CRP, and platelet. These factors have all been proven associated with the occurrence of HF. EMAT, defined as the period from the onset of the Q wave to the first peak of S1, reflecting the timing of electrical excitation and mechanical movement in the heart. Early studies have indicated that this timing is prolonged in HF patients. Li et al. (11) have reported that the heart sound and ECG signal index EMAT contributes to the diagnosis of ejection fraction <50%. Trabelsi et al. (29) found that HF patients exhibited higher EMAT and lower LVET compared to non-HF patients. The incidence of HF is linked to chronic systemic inflammation. We observed that elevated CRP levels are associated with an increased likelihood of HF occurrence. Burger et al. (30) similarly identified CRP as an independent risk factor for HF in patients with cardiovascular diseases. In summary, this research yielded results consistent with those obtained through traditional statistical analysis and ML-model studies, providing further validation of our findings.

Our findings have significant clinical implications. The performance of our final model was robust, indicating its potential utility in detecting early signs of HF in clinical settings. This could provide valuable support for implementing early risk management among patients with HF. Compared to traditional evaluation methods, the high sensitivity of ML-based detection tool could substantially improve HF early identification by reducing unnecessary hospitalizations and examinations, leading to significant time and cost savings. Our detection model boasted real-time applicability and scalability, as it can be automated and directly integrated into AI-PECG machines without requiring additional clinical data inputs (31). This suggested its practical utility in various healthcare settings, particularly in primary healthcare organizations where access to more invasive diagnostics may be limited. Additionally, the clinical decision support function of our HF detection model had immense practical value for non-professionals with limited experience in interpreting ECGs and PCGs. In clinical practice, non-professionals often encounter challenges in swiftly and accurately interpreting complex ECGs and PCGs. Our model addressed this issue by automatically analyzing ECG and PCG characteristics, delivering accurate HF risk prediction results promptly, and aiding in quick clinical decision-making. This capability had the potential to enhance the accuracy and efficiency of early HF detection, while also mitigating the risk of misdiagnosis or missed diagnoses attributable to imprecise judgments and human error.

Several limitations should be cautious to explain the findings. First, development of the HF detection model depended on features of ECGs and PCGs extracted from manufacturer-specific software. This implied that it requires retraining because of the variations in ECG and PCG signal pre-processing among different manufacturers when utilizing alternative software for signal processing. Second, although the selected features by data-driven technique had a positive effect on our model, a mixed strategy for feature selection needs to be future assessed. Third, despite analyzing data from three hospitals, our study encompassed only 1,017 patients, and the ML algorithm's performance could differ when applied to larger datasets with varying distributions of patient characteristics and across different institutions.

## Conclusion

In this study, we used the capabilities of ML to create a novel screening tool with high performance for HF, intended for clinicians’ use. Our findings indicated that integrating the analysis of PCG and ECG features markedly enhances the accuracy of HF screening, may surpassing traditional evaluation tools that rely solely on ECG or PCG features. Moreover, since this model aided early HF detection, it may further provide effective information on risk management strategies in HF patients.

## Data Availability

The raw data supporting the conclusions of this article will be made available by the authors, without undue reservation.
