# Supplementary figures and images for: Machine learning-based screening of heart failure using the integrated features of electrocardiogram and phonocardiogram: a multicenter study in China
Source: Front Cardiovasc Med. 2025 Nov 21;12:1613577. doi: 10.3389/fcvm.2025.1613577 (PMC12678375; doi:10.3389/fcvm.2025.1613577)

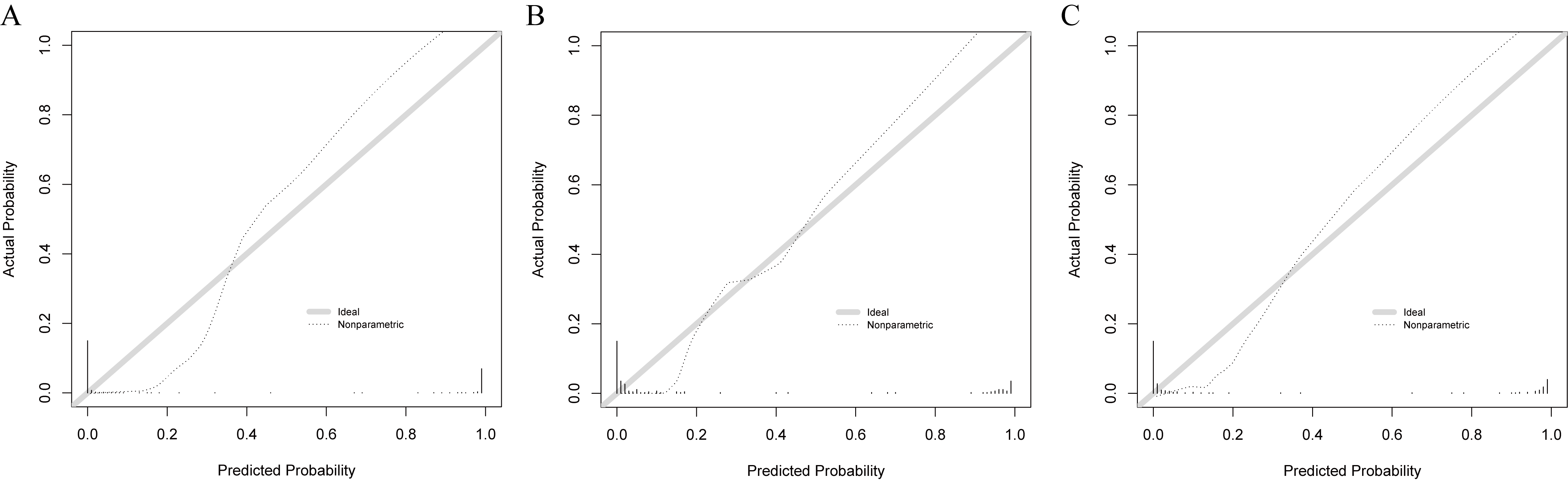

Supplement: Supplementary file 1 [file Image1.tiff]

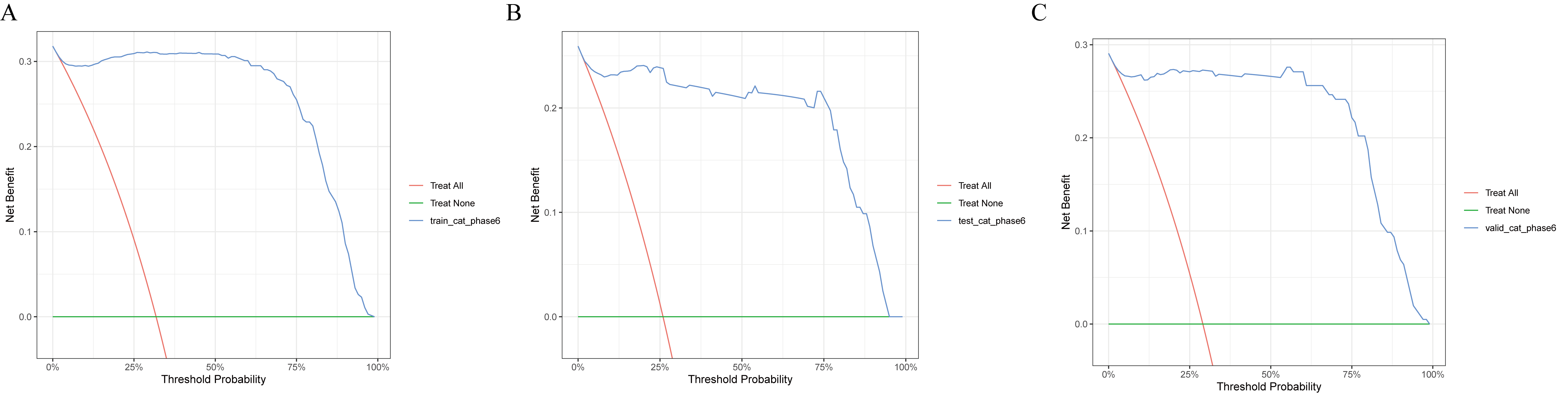

Supplement: Supplementary file 2 [file Image2.tiff]

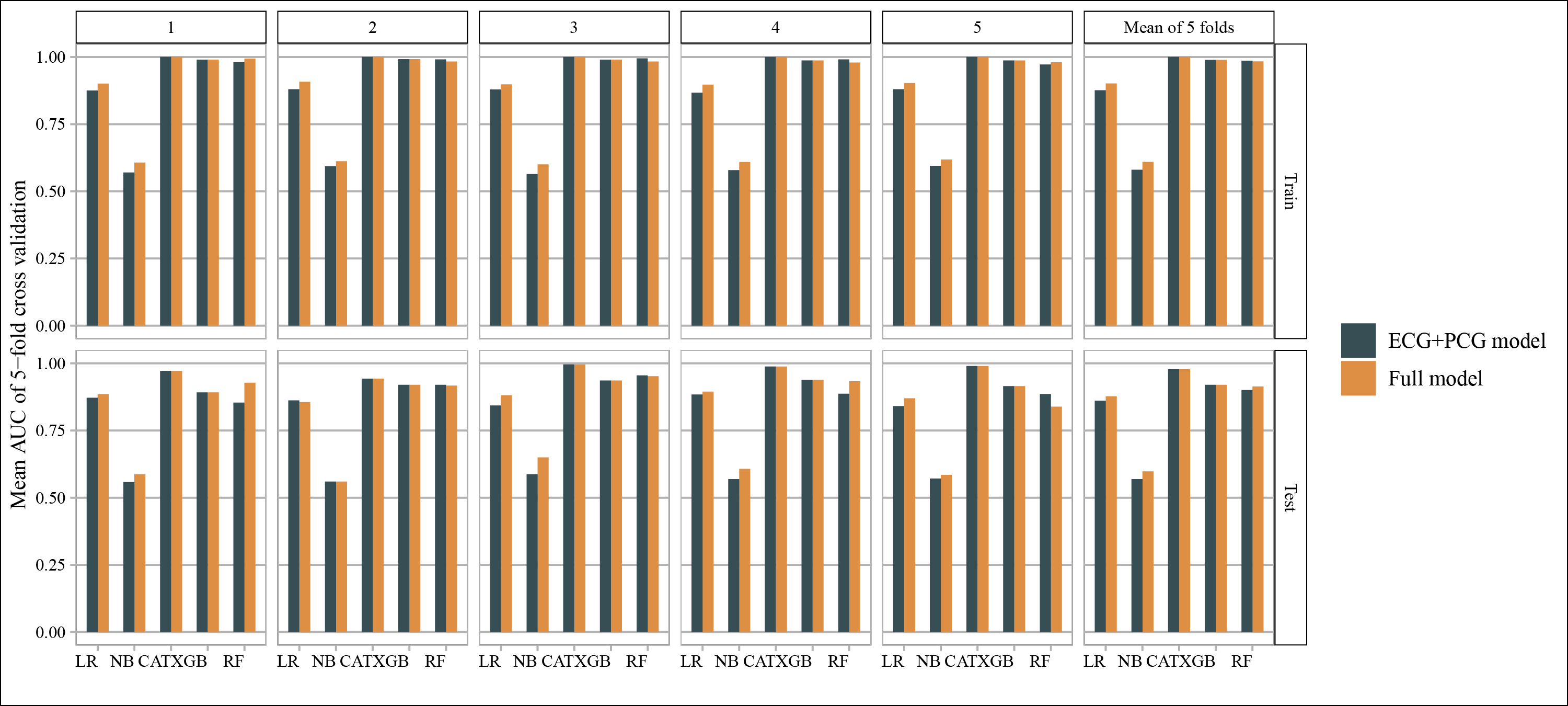

Supplement: Supplementary file 3 [file Image3.tiff]
